# A Review of Mushrooms as a Potential Source of Dietary Vitamin D

**DOI:** 10.3390/nu10101498

**Published:** 2018-10-13

**Authors:** Glenn Cardwell, Janet F. Bornman, Anthony P. James, Lucinda J. Black

**Affiliations:** 1 School of Public Health, Curtin University, Perth 6102, Australia; glenn.cardwell@postgrad.curtin.edu.au (G.C.); T.P.James@curtin.edu.au (A.P.J.); 2 School of Management, Curtin University, Perth 6102, Australia; Janet.Bornman@curtin.edu.au; 3 School of Veterinary Science, Murdoch University, Perth 6150, Australia

**Keywords:** vitamin D, mushroom, UV radiation, button mushroom, *Agaricus bisporus*, shiitake mushroom, *Lentinula edodes*, oyster mushroom, *Pleurotus ostreatus*

## Abstract

When commonly consumed mushroom species are exposed to a source of ultraviolet (UV) radiation, such as sunlight or a UV lamp, they can generate nutritionally relevant amounts of vitamin D. The most common form of vitamin D in mushrooms is D_2_, with lesser amounts of vitamins D_3_ and D_4_, while vitamin D_3_ is the most common form in animal foods. Although the levels of vitamin D_2_ in UV-exposed mushrooms may decrease with storage and cooking, if they are consumed before the ‘best-before’ date, vitamin D_2_ level is likely to remain above 10 μg/100 g fresh weight, which is higher than the level in most vitamin D-containing foods and similar to the daily requirement of vitamin D recommended internationally. Worldwide mushroom consumption has increased markedly in the past four decades, and mushrooms have the potential to be the only non-animal, unfortified food source of vitamin D that can provide a substantial amount of vitamin D_2_ in a single serve. This review examines the current information on the role of UV radiation in enhancing the concentration of vitamin D_2_ in mushrooms, the effects of storage and cooking on vitamin D_2_ content, and the bioavailability of vitamin D_2_ from mushrooms.

## 1. Introduction

Vitamin D stimulates the synthesis of the calcium transport proteins in the small intestine, enhancing the absorption of dietary calcium and thereby reducing the risk of osteomalacia in adults and rickets in children [1,2]. Adequate vitamin D is also important for muscle function and reducing the risk of falls in the elderly [3] and may help protect against some cancers, respiratory disease in children, cardiovascular disease, neurodegenerative diseases, and both type 1 and type 2 diabetes [4,5,6,7], although current evidence for non-skeletal benefits is inconclusive [8]. Although vitamin D is classified as a vitamin, it can be produced by the body in sufficient quantities when the skin is exposed to ultraviolet (UV) radiation from the sun [1]. If sunlight exposure is limited, dietary sources of vitamin D are required to maintain healthy circulating 25-hydroxyvitamin D (25(OH)D) concentrations. It is estimated that 1 billion people worldwide are vitamin D-deficient (25(OH)D concentrations ≤50 nmol/L), with prevalence of excess of 50% being commonly reported in population-based studies.

**Figure 1:**
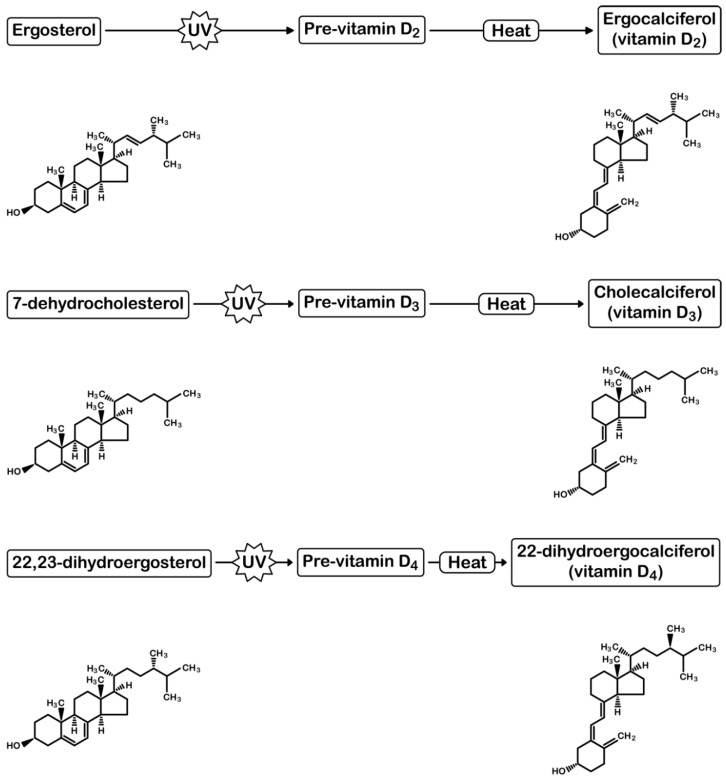
Structures of vitamin D_2_, D_3_, D_4,_ and their precursors. UV, ultraviolet radiation.

The two main dietary forms of vitamin D are D_2_, found in fungi and yeast, and D_3_, found in animals; lesser amounts of vitamin D_3_ and D_4_ are also found in fungi [9,10,11,12] (Figure 1). Few foods in the Western diet are a good source of vitamin D, with the best naturally occurring dietary source being oily fish. Some countries have liberal fortification policies, with foods such as milk, margarine, breakfast cereals, and juices, fortified with vitamin D [13,14]. Sun-dried and UV radiation-exposed mushrooms are a potentially important source of dietary vitamin D (as vitamin D_2_) [15,16,17]. Vitamin D-enhanced mushrooms are the only non-animal food product with substantial amounts of bioavailable vitamin D and, as such, have the potential to be a primary source of dietary vitamin D for vegans and vegetarians. 

This review addresses the potential of mushrooms as a good dietary source of vitamin D. We considered mushrooms exposed to different sources of UV radiation (solar radiation, UV fluorescent lamp, and pulsed UV lamp) to gauge the potential for increasing vitamin D_2_ content and to examine whether the amount of vitamin D_2_ generated was nutritionally significant. We focussed on the three most commonly consumed mushrooms worldwide: the button mushroom *Agaricus bisporus* (Lange) Imbach (30% of worldwide consumption), oyster mushrooms *Pleurotus* (Jacquin) Kummer (all species: 27% of worldwide consumption), and shiitake mushrooms *Lentinula edodes* (Berkeley) Pegler (17% of worldwide consumption), together comprising approximately three-quarters of all mushrooms consumed [18]. Studies of other edible mushroom species were included where context was needed or if there was very little information on the most popular mushrooms. This review is limited to English language publications and, for consistency and comparability, includes only those studies where vitamin D in mushrooms was measured using high-performance liquid chromatography. 

## 2. Requirements and Intake of Dietary Vitamin D

The recommended intake of vitamin D is 5–15 μg/day (200–600 IU) in Australia and New Zealand, depending on age [19], 15–20 μg/day (600–800 IU) in the USA [20], 15 μg/day (600 IU) as set by the European Food Safety Authority [21], 15–20 μg/day (600–800 IU) for Canadians [22], and 10 μg/day (400 IU) in the United Kingdom [23] (Table 1). 

Estimates of the dietary intake of vitamin D in the USA are 5–6 μg/day in adult males and 3.5–4.5 μg/day in adult females, although the intake of those taking vitamin D supplements may reach the Adequate Intake (AI) [24]. Canadian adults obtain an average of 5.8 μg/day from food, which includes vitamin D-fortified foods such as milk [25]. European intake of vitamin D is estimated to be 2–4 μg/day [13]. In the Irish population, the median intake of total vitamin D in adults is estimated as 3.5 μg/day, reaching 3.7 μg/day in those consuming vitamin D-fortified foods [26]. All these estimates are higher than the estimated adult dietary intake of 2–3 μg/day in Australia [27], where vitamin D fortification is more restricted. However, with improved analytical methods for vitamin D and its metabolites in food, the previously reported estimates of vitamin D intake in Australia have been disputed and may be as high as 4.3 μg/day from animal food alone, when including both vitamin D_3_ and its hydroxylated metabolite 25-hydroxyvitamin D_3_ (25(OH)D_3_) [28]. 

The discrepancy between actual and recommended vitamin D intakes indicates that dietary sources alone are unlikely to lead to adequate vitamin D status.

## 3. Vitamin D Metabolism in Mushrooms

There are five biological kingdoms: Animalia, Plantae, Fungi, Protista (e.g., algae), and Monera (e.g., bacteria) [29]. Mushrooms reside in the fungal kingdom, making them very different biological entities compared to plants and animals, despite being considered a vegetable from a culinary perspective. Unlike plants, mushrooms have high concentrations of ergosterol in their cell walls, playing a similar role as cholesterol in animals, i.e., strengthening cell membranes, modulating membrane fluidity, and assisting intracellular transport [30]. The presence of both ergosterol and vitamin D_2_ in mushrooms was first reported in the early 1930s [31].

When exposed to UV radiation, ergosterol in the mushroom cell wall is transformed to pre-vitamin D_2_, which is then thermally isomerised in a temperature-dependent process to ergocalciferol, commonly known as vitamin D_2_ [10,32]. Through a similar process, pro-vitamin D_4_ (22,23-dihydroergosterol) from mushrooms is converted to vitamin D_4_ [9]. All commonly consumed mushrooms have provitamin D_4_, making them a potential source of vitamin D_4_ if exposed to UV radiation [9]. In general, there is a positive correlation between D_2_ and D_4_ content in UV-irradiated mushrooms [9].

## 4. Vitamin D Content of Fresh Mushrooms

### 4.1. Fresh Wild Mushrooms

The recent interest in the vitamin D_2_ content of mushrooms began with the discovery that wild edible Finnish mushrooms, the funnel chanterelle (*Cantharellus tubaeformis* (Bulliard) Fries), sampled in late summer and early autumn provided 3–30 μg D_2_/100 g fresh weight (FW), compared with less than 1 μg D_2_/100 g FW in the button mushroom purchased from retail outlets [33]. Since then, large amounts of vitamin D_2_ have been found in wild funnel chanterelles (21.1 μg D_2_/100 g FW), *Cantharellus cibarius* (Fries) (10.7 μg D_2_/100 g FW), and *Boletus edulis* (58.7 μg D_2_/100 g FW) [34]. A smaller amount of vitamin D_2_ (1.5 μg/100 g FW) was reported in wild *Agaricus* species in Denmark [35].

### 4.2. Fresh Retail Mushrooms

Most fresh retail mushrooms sold in the UK, Europe, North America, Australia, and New Zealand, especially the button mushroom, are grown in atmospherically controlled growing rooms, then harvested and taken to market and retail outlets in refrigerated transport. As they are usually grown in darkness, the only time they are likely to be exposed to light is during picking under fluorescent lights, which usually emit little or no UV radiation. Hence, the vitamin D_2_ content of retail fresh button mushrooms sold around the world is commonly reported to be less than 1 μg/100 g FW [9,17,33,34,36,37]. As 100 g is considered to be a realistic serve of mushrooms (approximately three button mushrooms), a typical serve will provide negligible vitamin D_2_. The National Nutrient Database of the United States Department of Agriculture lists shiitake, white button, and oyster mushrooms as all containing less than 1 μg/100 g FW of vitamin D_2_ [38].

### 4.3. Fresh Mushrooms Exposed to Sunlight

When fresh button mushrooms are deliberately exposed to midday sunlight for 15–120 min, they generate significant amounts of vitamin D_2_, usually in excess of 10 μg/100 g FW [17,35,39,40], which approaches the daily requirement of vitamin D recommended in many countries (Table 1). However, the amount of vitamin D_2_ generated depends on the time of day, season, latitude, weather conditions, and exposure time. Since these mushrooms have a higher surface area to volume (hence, more ergosterol is exposed), sun-exposed sliced mushrooms produce more vitamin D_2_ than whole mushrooms from the same amount of UV radiation exposure [11,39,40]. At midday in mid-summer in Germany, the vitamin D_2_ content of sliced mushrooms was as high as 17.5 μg/100 g FW after 15 min of sun exposure and reached 32.5 μg/100 g FW after 60 min of sun exposure [40]. An unpublished Australian study on whole button mushrooms determined the vitamin D_2_ content after exposure to the midday winter sun in July in Sydney (personal communication, J. Ekman, Applied Horticultural Research, 12 August 2013). Sun exposure to a single layer of small button mushrooms was sufficient to generate 10 μg D_2_/100 g FW after 1 h, while large button mushrooms took 2 h to generate the same amount of vitamin D_2_.

### 4.4. Fresh Mushrooms Exposed to UV Radiation from Lamps

An efficient way to produce nutritionally relevant amounts of vitamin D_2_ is to expose mushrooms to specific, controlled levels of UV radiation via a fluorescent UV lamp or a pulsed UV lamp. Mushrooms will generate vitamin D_2_ in response to exposure to UV radiation both during growing phase and post-harvest; however, commercial growers use UV lamps post-harvest for practical reasons. Fresh mushrooms, when deliberately exposed to a UV radiation source post-harvest, will generate significant amounts of vitamin D_2_ often reaching 40 μg/g dried mass (DM) (*ca* 320 μg/100 g FW) [11,15,17,36,40,41,42,43,44,45]. The most effective wavelength to stimulate the production of vitamin D_2_ in mushrooms is UV-B radiation (280–315 nm) [43]. Some researchers have used UV-A (315–400 nm) [34,43,46,47] and UV-C (<280 nm) radiations [15,34,36,43,46,48,49], however UV-A radiation was not effective at increasing vitamin D_2_ concentrations in all cases [34]. 

In fresh shiitake mushrooms, ergosterol concentrations are highest in the gills, followed by the cap and stalk, with the gills having twice the concentration of ergosterol as the cap [46,50]. Subsequently, the gills of the shiitake mushroom generate more vitamin D_2_ when exposed to UV-B radiation than the cap or stalk [44], with the gills generating up to four times the vitamin D_2_ compared to the cap (22.8 μg/g DM vs. 5.2 μg/g DM) [46]. Whole oyster mushrooms have been shown to generate more than twice the amount of vitamin D_2_ than shiitake at the same UV exposure level [43,46]: when sliced and exposed to 60 min of UV-B lamp radiation, oyster mushrooms produced up to 140 μg/g DM [43,45,46,51,52]. Irradiation intensity was the most critical factor in determining vitamin D_2_ concentration: 90 min of exposure to UV-B radiation at 1.14 W/m^2^ at 28 °C were the optimal conditions for generating vitamin D_2_, producing 240 μg/g DM [52]. UV-B lamp irradiation has also been shown to increase vitamin D_4_ concentration in oyster mushrooms from 0 to 20 μg/g DM at 20 °C after only 30 min [51]. 

The influence of temperature on vitamin D_2_ production has not been investigated in detail, although two studies suggest that temperatures between 25 and 35 °C may be ideal for commercial purposes. One study showed that vitamin D_2_ production in whole oyster mushrooms increased from 152 μg/g DM to 178 μg/g DM as the temperature increased from 15 °C to 35 °C [52], while results from another study showed that the optimum conversion of ergosterol to vitamin D_2_ in shiitake mushrooms occurred at 35 °C and 78% moisture, producing ca. 50 μg/g DW [46].

As demonstrated in two studies, nutritionally relevant concentrations of vitamin D_2_ (10 μg/100 g FW) in whole mushrooms can also be effectively achieved with a commercial pulsed UV lamp within a very short time period of 1–2 s (3–6 pulses) [41,42]. In contrast, it can take several minutes to generate the same concentration of vitamin D_2_ using a UV fluorescent lamp. Therefore, pulsed UV radiation may be the most efficient method of increasing vitamin D_2_ concentrations in mushrooms. In button mushrooms, three pulses (1 s) of UV radiation generated 11.9 μg D_2_/g DM [42], and nine pulses (3 s) generated 20 μg D_2_/g DM [41]. The maximum concentration of vitamin D_2_ (27 μg/g DM) was reached after 12 pulses (4 s) [42]. The laboratories in both studies used similar pulsed UV lamp systems (Xenon Corporation), which produce a pulse of high-energy UV radiation (505 J/pulse) that is able to generate vitamin D_2_ deep within the ‘flesh’ of the mushroom. The mushrooms received either 1.1 J/cm^2^ per pulse [41] or 0.791 J/cm^2^ per pulse [42]. The concentration of vitamin D_2_ generated depends on the type and orientation of the mushrooms, whether they are sliced or whole, the distance from the lamp housing, the size of the mushroom, and the total number of pulses received. 

## 5. Dried Mushrooms Exposed to UV Radiation from Lamps

Commercial dried mushrooms have a much longer shelf life than fresh mushrooms, often with a ‘best-before’ date of 2–3 years after packaging. They have about 15% of the original weight of fresh mushrooms, making them cheaper to transport and, potentially, a cheaper source of vitamin D_2_. 

### 5.1. Sun-Dried Mushrooms 

Sun-drying is one method used for drying mushrooms in Asian countries. Analysis of vitamin D_2_ and ergosterol content of 35 species of dried mushrooms sold in China revealed they contained significant amounts of vitamin D_2_, with an average of 16.9 μg/g DM (range of 7–25 μg/g DM [48]). No details were provided on the method of drying, nor the time since the initial drying. The moisture content of the commercial dried mushrooms varied, although the majority contained 3–7% moisture.

### 5.2. Hot-Air Dried Mushrooms 

Mushroom collectors often pick mushrooms in the wild, dry them using a hot air method, then store the dried mushrooms for months or years. When mimicking this process, chanterelles (*C. cibarius*) collected from Swedish forests had vitamin D_2_ between 0.12 and 6.3 μg/g DM after being hot air-dried and stored in darkness for 2–6 years [53]. In a study of button, shiitake, and oyster mushrooms, the authors suggested that 60 °C is the optimum air-drying temperature post UV-B exposure, since obvious discolouration occurred above 60 °C [54]. When shiitake mushrooms were dried under laboratory conditions, the conversion of ergosterol to vitamin D_2_ was most efficient when the mushroom contained 70% moisture and received UV-B radiation (in the range of 290–320 nm in this study) for 2 h, producing 25 μg D_2_/g DM (ca. 200 μg D_2_/100 g FW) [50]. As the mushrooms dried in a desiccator over seven days, their ability to generate vitamin D_2_ decreased with the moisture content dropping from 70% to 30%, although vitamin D_2_ concentrations were still nutritionally significant at 30% moisture content (15 μg D_2_/g DM; ca. 120 μg D_2_/100 g FW). 

### 5.3. Freeze-Dried Mushrooms 

Mushrooms that have been freeze-dried will have close to zero moisture, resulting in 8–10% of the weight of the original mushroom (unlike dried mushrooms which still have a small water content, possibly 5%). Freeze-dried button, shiitake, and oyster mushrooms generated more vitamin D_2_ after exposure to UV-B radiation than hot air-dried mushrooms [55]. The authors suggested that the internal pore structures of the freeze-dried mushrooms facilitated the penetration of UV-B radiation. With no detectable vitamin D_2_ content before irradiation, freeze-dried oyster mushrooms generated 34.6 μg D_2_/g DM, shiitake 60 μg D_2_/g DM, and button mushrooms 119 μg D_2_/g DM after 30 min of exposure to radiation. Hot air-dried mushrooms with a moisture content of 6–8.3% produced 32–81 μg D_2_/g DM over the same time frame. 

Different variables (time of exposure, temperature, and exposure to UV-B radiation) can influence vitamin D_2_ production in button mushrooms that are freeze-dried and then powdered. For example, the ideal conditions for generating vitamin D_2_ from button mushroom powder were achieved by using a UV-B lamp (range 280–360 nm) with an irradiance of 1.36 W/m^2^ for 10 min at a temperature of 26 °C, producing 740 μg D_2_/g powder [56]. When freeze-dried, powdered shiitake mushrooms were exposed to 20 pulses from a Xenon RC 801 pulsed light system (UV range 190–700 nm), the concentration of vitamin D_2_ generated was 37 μg/g DM, while 60 pulses generated 62 μg/g DM [57]. 

## 6. Stability of Vitamin D_2_ in Vitamin D-Enhanced Mushrooms after Storage and Cooking

### 6.1. Storage

Analysis of the retention of vitamin D_2_ in both fresh and dried mushrooms exposed to UV radiation has mainly been done after refrigeration at 2–4 °C. Fresh button mushrooms stored at 2.2 °C showed a first-order kinetics decline in vitamin D_2_ concentration, with a predicted decline to a concentration of 1.75 μg/g DM at 14 days [58]. The vitamin D_2_ concentration in sliced button mushrooms dropped from 12 μg/g DM to 8–9 μg/g DM after 3–11 days storage at 3 °C [42]. Oyster and shiitake mushrooms stored at 4 °C showed a slight increase in vitamin D_2_ concentrations in the first 24 h of storage, before vitamin D_2_ level gradually reduced, over 10 days, to about one third to a half of the highest post UV-exposure level [59]. In one study, the vitamin D_2_ concentration in button mushrooms gradually increased from 3.5 μg/g DM to 8.1 μg/g DM during storage at 4 °C for six days, before dropping on days 7 and 8, while, for oyster and shiitake, vitamin D_2_ concentration dropped gradually over 10 days [59]. However, other studies did not show substantial vitamin D_2_ losses when mushrooms were refrigerated. There was virtually no degradation of vitamin D_2_ when button mushrooms were refrigerated at 4 °C for 8 days [36], or for 7 and 14 days [49]. Similar concentrations of vitamin D_2_ were found after one and four days in button mushrooms stored at 2.2 °C, equivalent to 70 μg D_2_/100 g FW [58]. Considered together, these studies suggest that UV-exposed fresh mushrooms will retain nutritionally relevant amounts of vitamin D_2_ when refrigerated for one week or less.

Three types of mushroom (button, shiitake, and oyster) exposed to a UV-B lamp and then hot air-dried, had relatively good retention of vitamin D_2_ up to eight months when stored in dry, dark conditions at 20 °C in closed plastic containers [55]. However, there was a steady loss of vitamin D_2_ during storage between 8 and 18 months. In the case of hot air-dried button mushrooms, vitamin D_2_ concentration decreased from 14.3 μg/g DM to 9.3 μg/g DM over eight months, then to 6.9 μg/g DM over the following 10 months. 

### 6.2. Cooking

Very few studies have investigated the effect of cooking on the concentration of vitamin D_2_ in vitamin D-enhanced mushrooms. Following 5 minutes of frying without oil, two types of wild chanterelle mushrooms retained at least 85% of their raw-state content of vitamin D_2_ concentrations after adjusting for water loss during cooking [60,61]. In button mushrooms with a vitamin D_2_ content of 19 μg/100 g FW, the retention of vitamin D_2_ after boiling in water for 20 min or oven-baking for 10 min, was 62–67%; for mushrooms pan-fried without oil for 5 min, the retention was again high, corresponding to 88% [61]. This indicates that the duration of cooking and the cooking method may be important factors in vitamin D_2_ retention in mushrooms.

## 7. Bioavailability of Vitamin D_2_ from Mushrooms

One of the earliest studies to determine the bioavailability of vitamin D_2_ was from wild chanterelles in the 1990s [62]. In 27 participants with a baseline mean serum 25(OH)D concentration of 38.5 nmol/L, vitamin D_2_ from mushrooms increased serum 25(OH)D concentrations as effectively as a vitamin D_2_ supplement after three weeks. Since then, the bioavailability of vitamin D_2_ from mushrooms has been demonstrated in both rats [36,63,64] and humans [10,65,66,67,68], and there is evidence that vitamin D_2_ from mushrooms supports bone health in animal models [47,64,69,70]. 

The bioavailability of vitamin D_2_ from mushrooms was assessed in a study of 30 healthy adults who were randomised to receive 2000 IU (50 μg) of supplemental vitamin D_2_, mushroom vitamin D_2_, or vitamin D_3_ for three months [10]. Vitamin D_2_ from mushrooms was as effective as supplemental vitamin D_2_ in raising and maintaining serum 25(OH)D_2_ concentrations. Similarly, a five-week study in adults with serum 25(OH)D (combined 25(OH)D_2_ and 25(OH)D_3_) concentrations less than 50 nmol/L showed that vitamin D_2_ from soup made from UV-B irradiated mushrooms improved vitamin D status as effectively as supplemental vitamin D_2_ [65]. However, another study providing UV-irradiated mushrooms as part of a meal for six weeks increased serum 25(OH)D_2_ concentrations in participants, whereas serum 25(OH)D_3_ concentrations decreased. Overall, there was no effect of the UV-irradiated mushrooms on vitamin D status [66]. Although research evidence indicates that vitamin D_3_ is more effective than vitamin D_2_ in raising the concentration of circulating 25(OH)D [71,72], it should also be acknowledged that vitamin D_3_ is not suited to many vegetarians and that a source of vitamin D_2_ may be preferred.

## 8. Conclusions

Mushroom consumption is increasing rapidly worldwide, with the production of mushrooms rising from 1 billion kg in 1978 to 27 billion kg in 2012 (an increase in per capita consumption from 0.25 kg to 4 kg) [18]. Since mushrooms provide nutritionally relevant amounts of B group vitamins and of the minerals selenium, potassium, copper, and zinc, they are a nutritious, low energy-dense food [73,74]. Currently, some larger commercial mushroom farms in the USA, Ireland, The Netherlands, and Australia expose fresh mushrooms to UV radiation, generating at least 10 μg D_2_/100 g FW; therefore, a 100 g serve would provide 50–100% of the daily required vitamin D to consumers. Exposing dried mushrooms to UV-B radiation can also generate nutritionally useful amounts of vitamin D_2_, although this practice is not widespread to date.

It is conceivable that UV-B radiation post-harvest (for fresh mushrooms) or post-drying (for dried and powdered mushrooms) could become standard commercial practice. Sunlight, regular UV lamps, and pulsed UV lamps have the capability to raise the vitamin D_2_ concentrations to nutritional significance, although pulsed UV lamps may be the most cost-efficient method for commercial production of vitamin D-enhanced mushrooms, because of the low exposure time (often in 1–3 seconds) to achieve at least 10 μg/100 g FW. There is minimal discolouration in mushrooms after pulsed UV treatment, possibly due to the small exposure time of less than 4 seconds [42]; however, there are many reports of surface discolouration of mushrooms after longer exposures to UV radiation from UV fluorescent lamps [15,34,36,44]. Since consumers may be deterred by mushrooms discolouration, pulsed UV treatment is likely to be preferred by commercial growers.

Vitamin D-enhanced mushrooms contain high concentrations of vitamin D_2_, which is bioavailable and relatively stable during storage and cooking. Therefore, consumption of vitamin D-enhanced mushrooms could substantially contribute to alleviating the global public health issue of vitamin D deficiency. Further research is warranted to determine the optimal level of UV radiation required to produce a nutritionally useful amount of vitamin D_2_ in mushrooms, along with optimal storage conditions and cooking methods. The physiological benefits of mushroom-derived vitamin D_2_ compared with solar-derived vitamin D_3_ also require further investigation.

## Figures and Tables

**Table 1:** Examples of recommended daily intakes of vitamin D (μg/day) across different regions.

	Age (Years)				
	**1**–**18**	**19**–**30**	**31**–**50**	**51**–**70**	**71+**
United States of America ^a^	15	15	15	15	20
Canada ^b^	15	15	15	15	20
United Kingdom ^c^	10	10	10	10	10
Europe ^d^	15	15	15	15	15
Australia and New Zealand ^e^	5	5	5	10	15

^a^ USA, Recommended Dietary Allowance (RDA) [20]; ^b^ Canada, Adequate Intake (AI) [22]; ^c^ UK, Reference Nutrient Intake (RNI) [23]; ^d^ Europe, AI [21]; ^e^ Australia and NZ, AI [19].
